# Salient beliefs about modifiable risk behaviours among patients living with diabetes, hypertension or both: A qualitative formative study

**DOI:** 10.4102/phcfm.v14i1.3327

**Published:** 2022-09-09

**Authors:** Prosper Lutala, Peter Nyasulu, Adamson Muula

**Affiliations:** 1 Department of Family Medicine, Faculty of Medicine and Oral Health, Kamuzu University of Health Sciences, Blantyre, Malawi; 2 NCD-BRITE Consortium, Faculty of Public and Global Health, Kamuzu University of Health Sciences, Blantyre, Malawi; 3 Department of Global Health, Division of Epidemiology and Biostatistics, Faculty of Medicine and Health Sciences, Stellenbosch University, Cape Town, South Africa; 4 Division of Epidemiology and Biostatistics, School of Public Health, Faculty of Health Sciences, University of the Witwatersrand, Johannesburg, South Africa; 5 Department of Public Health, Faculty of Public and Global Health, Kamuzu University of Health Sciences, Blantyre, Malawi; 6 African Center for Public Health and Herbal Medicine (ACEPHEM), Faculty of Public and Global Health, Kamuzu University of Health Sciences, Blantyre, Malawi

**Keywords:** diabetes, hypertension, lifestyle, Malawi, Mangochi, modifiable behaviours, noncommunicable diseases, risk, salient belief, theory of planned behaviour

## Abstract

**Background:**

Although there is evidence of the key role played by focusing on local knowledge in designing appropriate interventions regarding modifiable risk behaviours among patients living with diabetes and hypertension in Mangochi (and Malawi), little is known about local salient beliefs.

**Aim:**

With a focus on the theory of planned behaviour as a theoretical lens, this study aimed to identify salient beliefs about modifiable risk behaviours among patients with diabetes, hypertension or both in Mangochi, south-eastern Malawi. Specifically, the objectives were to identify advantages and disadvantages (behavioural salient beliefs), people who approve or disapprove (normative salient beliefs) and enablers and barriers (control salient beliefs) for measures to change modifiable risk behaviours among patients with diabetes, hypertension or both in Mangochi, Malawi.

**Setting:**

A hypertension diabetes clinic at Mangochi District Hospital, south-eastern Malawi.

**Methods:**

A formative qualitative study of a quasi-experimental trial was conducted among 25 patients, purposefully sampled, who were living with diabetes, hypertension or both at Mangochi District Hospital in February 2019. Researchers conducted in-depth interviews with patients using an interview guide informed by the theory of planned behaviour’s elicitation interview guide. Thematic content analysis was used to identify emerging themes.

**Results:**

A total of 25 participants were recruited, of which 12 (48%) were living with diabetes. Five thematic areas emerged from this analysis: physical and psychological fitness, social disconnection, perceived support systems, perceived enablers and perceived barriers to change.

**Conclusion:**

Appropriate words for each salient belief were identified. Future researchers should use the identified salient beliefs when designing interventions based on the theory of planned behaviour in diabetes and hypertension.

**Contribution:**

The paper adds to the body of knowledge informing the use of theory of planned behavior in addressing modifiable risk factors among practitioners, specialists and academics in primary care and Family Medicine in the field of noncommunicable diseases in Mangochi Malawi and beyond.

## Introduction

Diet choices as a type of behavioural risk factor have an effect on diabetes onset and control.^1,2^ In 2017, unhealthy diets led to 11 million deaths and 255 million disability-adjusted life years (DALYs) globally,^3^ while low intake of fruits alone claimed 2 million people and 65 million DALYs during the same period.^3,4,5^ Furthermore, a 2018 Physical Activity Guidelines Advisory Committee Scientific Report combined with a systematic review of articles from 2017 to 2018 showed a strong correlation between sedentary behaviour and increased all-cause mortality risk, incidence of diabetes and cardiovascular diseases, as well as cancers of the colon, lung and endometrium.^6^ This correlation between lifestyle risk factors and disease is true for all modifiable risk factors: unhealthy diets, harmful alcohol intake, smoking, physical inactivity, etc. Although there is high prevalence of risk factors in Malawi,^7^ addressing these risk factors can result in a lower risk of diabetes in nonobese subjects^8^ and improvement in the conditions or risk factors of others. For the sake of this study, noncommunicable diseases (abbreviated henceforth as NCDs) will refer to diabetes and hypertension.

## Theoretical framework

**FIGURE 1:**
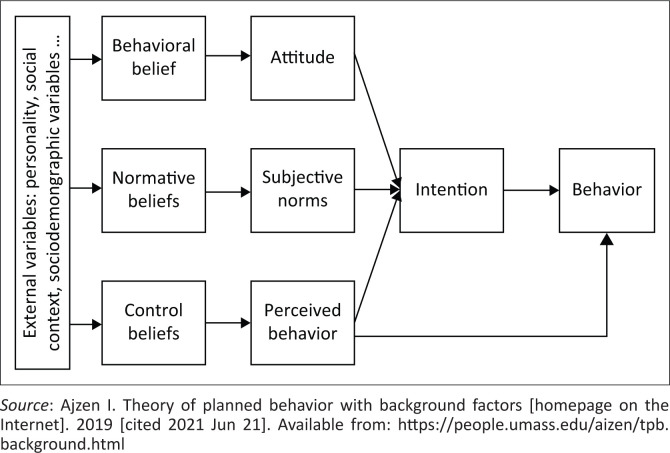
Theory of planned behaviour.^27^

The theory of planned behaviour (TPB) (Figure 1) posits that intention to change a behaviour is determined by a person’s attitude towards the behaviour (perception of positive and negative consequences associated with carrying out the behaviour), the subjective norm (perception of social pressure to carry out the behaviour) and the perception of control (perception of facilitators or barriers) over the behaviour. This perception acts on behaviour through intention to perform the behaviour. Perception of control can also directly impact the behaviour without going through the intention^9,10,11^ (see framework of the TPB in Figure 1).

The TPB is popular and frequently employed in social psychology,^12,13,14^ owing to its clear constructs through which behaviour is hypothesised to operate.^15^ The project chose this theory for this study because of its ability to predict and explain people’s behaviours and intentions^16,17,18^ and its use in developing behaviour change interventions.^18^

Several TPB studies have been conducted to examine behavioural, normative and control beliefs associated with some behaviour such as physical activity, eating fruits and vegetables, smoking tobacco and drinking alcohol.^19,20,21^

While studying whether the TPB significantly explained the relationship between intentions and behaviour over a 1-month period in a sample of 183 students in Grades 7–9 who completed a questionnaire based on TPB constructs at two time points, Murnaghan et al. in 2010 found that the attitude–intention relationship was moderately large for fruit and vegetable consumption and small to moderate for being smoke-free.^19^ Perceived behavioural control had a large effect on being smoke-free and a moderately large effect for fruit and vegetable consumption and physical activity. Intention had a large direct effect on all three behaviours.^19^ A second study was carried out in which the TPB aimed to encourage parents to include more fruit, vegetables and whole grains in the lunches packed for their preschool children.^22^ Evaluated measures included knowledge, behavioural control, perceived behavioural control, subjective norms and intention; the intervention component revealed an increase in vegetables and whole grains in the lunches parents prepared for their children.

To the best of the authors’ knowledge, there are no recent studies based on the TPB identifying specific local words or concepts which could be used to translate modal beliefs regarding alcohol use, smoking, unhealthy diets and physical inactivity among patients with diabetes, hypertension or both in Mangochi, south-eastern Malawi.

## Research objectives

The study’s aim was to identify modal beliefs about eating healthy diets, exercising, excessive alcohol consumption and smoking with specific objectives:

- To identify the advantages and disadvantages (behavioural salient beliefs) of modifiable risk behaviour change
- To identify people who approve or disapprove (normative salient beliefs) of modifiable risk behaviour change
- To identify enablers and barriers (control salient beliefs) regarding measures to change and modifiable risk factors among patients with diabetes, hypertension or both in Mangochi, south-eastern Malawi.

From these objectives, the research questions was: what are the behavioural, normative and control salient beliefs about measures to change behaviour in patients living with diabetes, hypertension or both regarding unhealthy diet, smoking, harmful alcohol consumption and physical inactivity?

This elicitation study will help to generate appropriate local words which will be included in an adapted questionnaire for later use in using the TPB for related interventions such as prediction or evaluation of intention, efficacy, cost-effectivess and behaviour related to the given risk factors.

## Methods

### Study design

This is a one-phase formative qualitative study based on Fishbein and Ajzen’s recommendation of undertaking a belief elicitation study before any intervention using the TPB, carried out among 25 participants selected from a NCD clinic in Mangochi, Malawi.^23^ The current study was nested in a research project on feasibility and efficacy of brief behavior change in lifestyle, which was itself part of an implementation project.

### Setting

Mangochi district is located in the south-east of Malawi, along Lake Malawi and bordering Mozambique. The district covers an area of 6273 km² and has a population of 1 224 716.^24^ In the Malawian context, the people in the Mangochi district are relatively poor. Farming is the major source of income, with tobacco being the major cash crop. So far, NCD clinics are present in three facilities: the Mangochi district hospital, St Martins Mission Hospital and Monkey Bay Community Hospital. The first two are government-owned, providing free care and receiving drugs and other supplies from the central government. The last is a mission fee-paying hospital, which purchases all the drugs and commodities to run the clinics. The overall quality of care provided locally is suboptimal, and there is very little difference between care provided throughout the primary and secondary levels of the health system.^25^ The clinic’s focus remains solely on diabetes and hypertension, despite other conditions listed on the monitoring and evaluation (M&E) tools. The NCDs programmes are affected nationally by constraints such as shortage of staff and lack of training, drugs and supplies, with frequent stock-outs and a lack of guidelines and training materials.^26^

### Sampling and recruitment

This study included adults with clinically stable diabetes, hypertension or both, aged 18 years and above, who were coming for refill visits at the NCD clinics at Mangochi District Hospital. The population of younger patients, who are more likely to suffer from diabetes mellitus type 1 (T1-DM), which is more insulin-dependent than lifestyle-dependent, was excluded from this study.

The research team used a purposeful sampling, targeting participants who were attending the NCD clinics for their ability to provide rich information.^28^ Participants were chosen and assigned to attend an interview using a questionnaire specific to their most prevalent modifiable risk behaviour: unhealthy eating, smoking, physical inactivity or harmful alcohol consumption. Twenty-one participants were selected using the snowballing method.^29^ This method involves patients being asked by the research team to suggest fellow clinic patients who have the same ability to contribute salient beliefs about the risk factors (the phenomena under study here).^28,30^

Up to 25 participants were recruited, at which time the saturation of narrative data was recognised and new ideas were not being generated. Furthermore, 25 participants are the minimum sample size required for studies based on previous recommendations in elicitation studies based on the TPB.^31^

### Data collection

In-depth interviews were held using open-ended questions for each participant between December 2019 and February 2020 with the 25 participants in the consultation room at the end of the clinic visit, using an interview guide. The median duration of the interview was 36 min (interquartile range [IQR] = 5). The guide was designed to explore key concepts about behaviours: advantages and disadvantages, their easy or difficult character, individuals who would approve or disapprove of behaviours and circumstances, as well as which would make one engage or disengage in the behaviour.^32^ Interviewers were trained to identify themes to extract and use during the ongoing interview to further elaborate on ideas which were progressively emerging.

The interview was mostly in Chichewa (the national language in Malawi), with some facilitation or probing in Chiyao (the most widely spoken local language in Mangochi) for clarification and to attract the interest of some native Chiyao speakers. The behaviours explored were eating three portions of fruits and vegetables daily, engaging in at least 30 min of rigorous or moderate physical activity per day for 5 days per week or more, stopping smoking and reducing alcohol intake (no more than two standard drinks for men or more than one for women per day).

For the questionnaire used in data collection for the three salient beliefs (Appendix 1).^32^

The digitally recorded interviews were conducted by two trained research assistants (a nurse and a clinical officer, both Malawian, fluent in Chichewa and Chiyao), who were assisted by the principal investigator (a family physician, non-Malawian, who can understand and speak Chichewa professionally but is illiterate in Chiyao). Both have previous experience as research assistants participating in studies conducted in the district, including qualitative research. Assistant researchers were also actively working in the hospital but without any assignment in the diabetes–hypertension clinic at that time. All have been actively involved in the development of this research project with assistance in questionnaire designing and piloting.

Sociodemographic data including age, sex, address, condition (hypertension, diabetes or both) and number of years with the condition(s) were recorded prior to the interviews.

### Data analysis

The analysis of data was carried out using thematic content analysis. Data were transcribed in Chichewa by a professional clerk at the university before being translated into English by a graduate in English literature, fluent in Chichewa, Chiyao and English. A second researcher back-translated the data to Chichewa. Both the initial and the second Chichewa versions of the transcripts were compared to ensure that no distortion of data took place through the translation processes.

**FIGURE 2:**
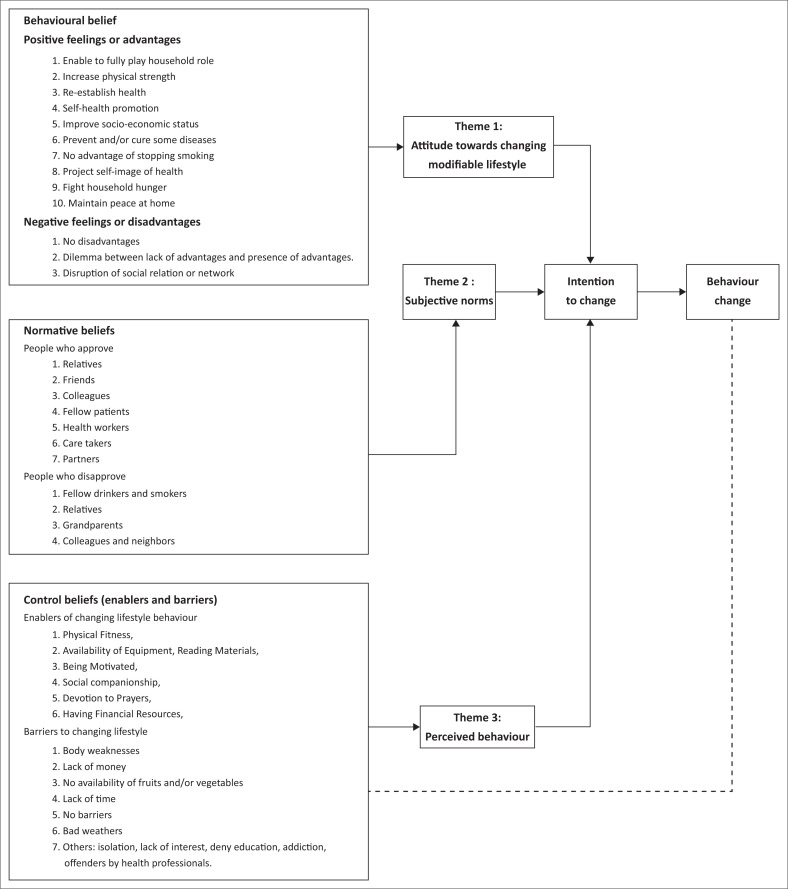
Model of salient beliefs about modifiable behaviour among patients attending a NCD clinic.

After collection, the data were coded using the words of the participants and analysed before identifying themes and patterns that best described the patients’ salient beliefs regarding the four behaviours. Categories were put in Figure 2 under their corresponding TPB constructs. A consolidation of findings from each participant was performed in a group, and the recurrent themes were highlighted for this data presentation. For the sake of consistency, the list of categories and concepts were compared between four team members and reconciled through discussion whenever discrepancies were observed. The principal researcher also conducted a check to ensure that the meaning in the transcript had not been distorted during the process of coding or during subsequent phases. For the sake of this research, extraction of up to five of the most common beliefs from the long list of participants’ answers were extracted. This was in line with research of Ajzen, who recommends that only the first five salient beliefs should be kept without compromising their validity.^33^ At times, the list of salient beliefs was fewer than four. Extraction categories (illustrated by quotes) for each key salient belief for illustration in the results section are presented. Afterwards, different categories were grouped in diverse themes.

### Trustworthiness of the data

To ensure trustworthiness of the data, credibility, dependability, conformability and transferability were considered. Research began by piloting the questionnaire before conducting research and illustrating the findings with direct quotes to ensure the study’s credibility and dependability. Conformability was achieved by triangulating data through collecting information from multiple cadres (nurses, clinicians), types of informants (patients and healthcare providers), multiple data collection approaches (focus groups and key informant interviews).^34^ The study considered transferability of the study, as views came from individuals with a variety of experiences, drawn from those living with both diseases (diabetes, hypertension and hypertension–diabetes comorbidities), coming from different areas (semi-urban and rural).^34^

### Ethical considerations

Ethical clearance was granted by the Kamuzu University of Health Sciences’s College of Medicine Research and Ethical Committee (COMREC, reference number P.03/20/2971). Data were collected anonymously after local authorisation and participants signing a written informed consent form. We ensure confidentiality of data throughout the study.

## Findings

### Sociodemographic characteristics

A total of 25 participants were recruited into the study, of whom 16 (64%) were female. The median age was 55 years, with the youngest participant being 20 and oldest 75 years. Of the participants, 6 (24 %) had hypertension, 12 (48%) DM and 7 (28%) had both hypertension and DM. The median duration since the onset for diabetes and hypertension was 72 months (IQR = 5 months). Regarding modifiable behaviours, participants were distributed as follows: two used alcohol (8%), three were smokers (12%), seven were physically inactive (28%) and 13 had unhealthy diets (52%).

### Themes

The themes (with their corresponding categories as expressed by participants) extracted from data analysis were physical and psychological fitness (normalise the body, neutral state of body, free from minor ailments, well-controlled diabetes or hypertension); being socially disconnected (being abandoned); perceived support system (close relatives and friends, fellow patients and past victims of unhealthy behaviour) or non-supportive system (fellow drinkers or smokers, healthy people, vendors of alcohol or tobacco and unwilling family members); perceived enablers (physical fitness, availability of reading materials, availability of equipment, high motivation, having a source of support and disclosure of the condition) and perceived barriers (addiction, lack of money, refractory to advice and busy schedule). Details of the themes, categories and codes attached to their respective constructs are in the study model portrayed in Figure 2. However, the themes, categories and their definitions are portrayed in Table 1-A1.

#### Physical and psychological fitness

A participant living with diabetes and two other participants living with both hypertension and diabetes perceived that changing behaviour can contribute to normalising the body, reverting it to normal structure and function, and in doing so, it may prevent diseases and conditions. This was echoed by a female participant, who said:

‘[*S*]topping smoking is the way to go. [*T*]he body felt going back to normal afterwards.’ (Participant 8, a 43-year-old woman living with diabetes)

Another participant felt that the key to having a healthy body is by becoming physically and emotionally fit through a well-balanced healthy diet. He emphasised the role of food for patients with diabetes in this way:

‘[*B*]eyond these diabetes and hypertension we are suffering from, in general, healthy diet gives you a well healthy body, a well-balanced one …’ (Participant 4, living with diabetes, 52-year-old man)

Rather than considering behaviour change in a more abstract sense, patients expressed physical activity with practical phrases such as ‘a change …’, ‘start of work’, ‘start of entertainment’, ‘asymptomatic state’ and even ‘household’s role changing or increasing’. A participant reported that physical activity adjustment resulted in several observed changes. Since he started doing physical activities, he is able to play with the children at home:

‘I have seen change in my body since I have started exercising, cycling daily and playing with children, a big change in me.’ (Participant 4, a 52-year-old man living with diabetes)

His contribution to routine work or his role in the household has improved:

‘I am really free. [*E*]ver since, I can work at home, in the fields, do my small business …’ (Participant 4, a 52-year-old man living with diabetes)

He has also seen a reduction in the frequency with which he experienced several minor ailments, and he has a feeling of well-controlled diabetes:

‘The recurrent running nose, fatigues, headaches and other small ailments have stopped …’ (Participant 4, a 52-year-old man living with diabetes)

‘I think of diseases only when taking my antidiabetics tablets … I am really free.’ (Participant 4, a 52-year-old man living with diabetes)

Regarding the perceived lack of side-effects of behavior change a third group of participants denied any harm from changing modifiable behaviours. Their perceptions were expressed as ‘no disadvantages or uncertainty’. An elderly participant did not find any complication after initiating physical activities in his life:

‘[*C*]annot imagine any problems after incorporating physical activity in my daily life … none really.’ (Participant 16, a 51-year-old woman living with diabetes and hypertension)

Others had no knowledge of downsides to stopping harmful behaviours. A middle-aged man living with hypertension denied any negative effects related to quitting smoking, saying:

‘Disadvantages of stopping smoking … As such … I don’t know.’ (Participant 17, a 39-year-old man living with hypertension)

Another participant echoed him, emphasising a lack of harm after stopping smoking or drinking alcohol. He stated:

‘I wonder what can be the disadvantage of stopping smoking or reducing alcohol … I don’t really know.’ (Participant 21, a 49-year-old man living with hypertension and diabetes)

#### Disrupted social network

One of the participants foresaw losing friends if he stopped (or even decreased) his alcohol intake. He expressed his feeling of disconnection in this way:

‘[… *F*]ind yourself alone […]’ (Participant 21, a 49-year-old man living with hypertension and diabetes)

Another participant added that men were most at risk for loneliness, which he expressed in this way:

‘[… *A*]lcohol drinking is really socialising, once off … ah, my doctor, you find yourself alone, you lose friends. You see, n’ male […]?” (Participant 21, a 49-year-old man living with hypertension and diabetes)

#### Significant others as support source

The sources of support for behaviour change were quite diverse, ranging from relatives, friends, fellow patients and caretakers to the patients’ workmates. The reasons for approval may depend on several factors, including the effect of the patient’s behaviour on the person approving of that behaviour.

Talking about his smoking habit, rather than describing the different players who would discourage him from changing his behaviour, a 27-year-old man stressed the support he would get from his direct social network in stopping smoking. About smoking, he said:

‘My dad, my sisters and even my other relatives and friends will really support me to quit smoking […]’ (Participant 5, a 27-year-old man living with diabetes)

With regard to exercising, another participant added this:

‘[*M*]y brothers, sisters and nieces, alongside my husband will approve for sure my busy programmes of exercising.’ (participant 20, a 31-year-old woman living with hypertension)

A second group considers their fellow patients (during the waiting time at the clinic) as source of support in whatever modifiable lifestyle behaviour research would like to improve. A middle-aged woman said this:

‘[*F*]ellow patients during our discussion at the clinic’s waiting area will be the first to advise me to stop adding salt to my food and taking oil foods and increase consumption of salads and fruits.’ (Participant 13, a 45-year-old woman living with diabetes)

Finally, other respondents said that individuals who have been negatively affected by some modifiable behaviours such as alcohol intake would be a source of strong support for them. To this end, one respondent said:

‘[*M*]y wife has really suffered with my drinking […] at times we fight, quarrel and even chase each other on the streets in our neighbourhood. Yes, I foresee her to be very supportive at time now … [*in changing behaviour*].’ (Participant 7, a 44-year-old man living with diabetes)

#### Perceived non-supportive groups of people

Those whom most participants perceived as non-supportive included: fellow drinkers and smokers, friends, grandparents, sellers (of products involved in an unhealthy behaviour) and healthy people. In few case, participants could not identify anyone. Doubting his chances of stopping, a participant expressed:

‘[*F*]ellow smokers will totally disapprove any attempt of stopping smoking I can undertake.’ (Participant 5, a 27-year-old man living with diabetes)

Another stated:

‘[F]ellow drinkers will be the first barrier to overcome my health problem. […] Any suggestion to stop [*drinking alcohol*] will be rejected.’ (Participant 18, a 42-year-old living with diabetes and hypertension)

From time to time, family members will be an obstacle to patients with hypertension or diabetics in changing lifestyle behaviours because of lack of knowledge, with or without a sense of sympathy or compassion:

‘[*G*]randparents disapprove any novelty in full diets or even fruits in our houses, even among their children and grandchildren.’ (Participant 3, 25-year-old man living with diabetes)

Relatives’ feelings of concern, coupled with ignorance about diabetes or hypertension, led a participant to make the following statement about the benefits of exercising:

‘[*R*]elatives cannot accept me to start jumping and running, as they consider my high blood pressure to worsen with such vigorous body movement.’ (Participant 8, a 42-year-old woman living with hypertension)

#### Perceived enablers

**Facilitators of behaviour change:** Respondents alluded to diverse situations that may contribute to making behaviour change much easier. Those situations include: being physically fit, availability of equipment, availability of reading materials, motivation, having a source of support such as a helper (guardian) and disclosure of the condition to surrounding colleagues, extended family members and neighbours. One said this:

‘Being known [*by the above people*] already diabetic patients will assist to be understood when it comes to change your diet.’ (Participant 4, a 52-year-old man living with diabetes)

Many patients expressed that the availability of the instruments of physical activity, appropriate foods or any other equipment or materials involved in changing behaviour is the first step to enable change:

‘[*H*]aving those fruits and vegetables right there in my garden is a step forward in adopting the new advice in diets we receive from you …’ (Participant 19, a 51-year-old woman living with diabetes and hypertension)

‘Having equipment for good exercising is paramount to keeping these physical activities going; otherwise, they turn to be boring.’ (Participant 16, a 51-year-old man living with diabetes and hypertension)

A spiritual attitude was also mentioned by a participant as a way to facilitate change of behavioural lifestyle.

Turning to God in stopping the consumption of locally distilled alcohol was expressed through these words:

‘[… *A*]s I have turned to devotion in prayer, I know I will defeat the *kachasu* [*locally brewed spirit*] and other problems alike’ (Participant 18, a 42-year-old man living with diabetes and hypertension)

#### Perceived barriers to modifiable lifestyle behaviour change

Participants felt that behaviour change would be more challenging for those who were already addicted and weak, those without equipment or finances, those who were resistant to advice and those who were lonely or busy with many businesses. That addiction may interfere with the willingness to change behaviour and make it more challenging, one participant remarked:

‘For those addicted, they may need hundredfold effort to come out of it [*smoking*] …’ (Participant 8, a 43-year-old man living with diabetes)

The behavioural change, although it is taught free of charge, requires some resources for its successful implementation. A group of participants reiterated that behavioural change requires someone living with hypertension to have a clear vision and to have means. To express his financial constraints, one participant emphasised:

‘I have been told about diets, physical activity, alcohol and smoking by nurses and doctors for several years now … But my chronic lack of money these days makes it difficult to adopt appropriate diets, you know …’ (Participant 23, a 51-year-old man living with hypertension)

Another added:

‘[*L*]ack of vision makes it hard to stop alcohol consumption” (Participant 18, a 42-year-old man living with diabetes and hypertension)

Some behavioural change, such as increasing physical activity, is aided by a companion for support and encouragement. Without this, sustainability can be challenging. One responded expressed in this way:

‘[*D*]oing exercises alone [*without a companion*!] … it makes it difficult to sustain …’ (Participant 16, a 51-year-old woman living with diabetes and hypertension)

There was a general feeling among participants that behavioural change is closely related to changes to the taste of food, as salt is restricted. As much as one participant described his adherence to health diets, he expressed the negative feeling he endures each time the ‘new meal’ is served:

‘[*O*]ne disadvantage of following these foods without salt, oils and alike […] is most of the times for me, a lack of taste […] food is tasteless, difficult to swallow.’ (Participant 14, a 49-year-old man living with hypertension and diabetes)

## Discussion

### Key findings

This study suggests three major themes and several major categories of description of salient beliefs of patients with diabetes and hypertension regarding modifiable health behaviours. The first theme was physical and psychological fitness and social disconnection (perceived advantages or disadvantages of change) with four categories included: fitness of the body, sense of balanced physical activity, absence of diseases and lack of any disadvantages to change. The perceived support systems (social network support) are made by friends, relatives, partners and colleagues of patients. The third theme was perceived enablers and perceived barriers to change (or sources of support) with financial, human and equipment categories.

#### Physical and psychological fitness and social disconnection

The perception of advantages and disadvantages of behavioural changes to fitness of the body, sense of balanced physical activity (or lack thereof) and lack of diseases were reported by study participants. The sense of a balanced body was expressed by participants because of the feeling of a well, healthy body after stopping smoking and the ability to play with children, etc. Furthermore, positive changes in a few symptoms or subjective feelings (such as dizziness, headache, polyuria and dry mouth) were among the first expressed feelings following behavioural change. An early study also showed similar beliefs among participants: social engagement, relaxation, stress relief, enjoyment of alcohol, regaining or maintaining sense of self and stress relief.^35^

Salient beliefs in a positive or negative direction depend also on the context or targeted population in which the study was conducted. For example, unlike in our study where the disadvantages of alcohol focused on social components, an elicitation study among pregnant women that aimed to list the disadvantages of drinking alcohol showed the following setbacks: a previous study showed that in addition to normalising behaviour risk factors, motivational interviewing also has a perceived effect on the normalisation of biological markers.^36^ In this study, most participants expressed a positive attitude towards changing modifiable lifestyle behaviours, as behaviour change will ‘reverse the body to its normal structure and function’ or ‘prevent diseases and other conditions’. Similarly, improved, objective measures used to evaluate the effect of behaviour change included measurement of blood pressure and fasting blood sugar readings. Additionally, cessation of subjective feelings and symptoms such as dizziness, headache, polyuria and dry mouth as well as a sense of well-being or balance are often the first identified effects of the changes.

These positive links between the adoption of new behaviours and the perception of a positive change in the body need to be exploited by those designing promotion and preventive messages aimed at people living with diabetes in order to increase their likelihood of adopting new healthy lifestyles. It has been shown that a positive attitude towards a given health behaviour is the main ingredient for patients to adopt that behaviour.^37^

#### Perceived supportive system

Concerning participants’ perceptions regarding the normative salient beliefs, this study points out the role of networks of friends, relatives, partners and co-workers (colleagues), which appeared to be the key behavioural modifiers underscoring the influence played in patients’ continued care. A study aiming to elicit students’ salient beliefs about binge drinking found that being approved by their sports team (i.e. normative beliefs)^38^ was promoting drinking behaviour. This may mean that the proper use of this network can contribute to reversing the behaviour. The support received from this network plays a significant role in the adoption of change. It has also been demonstrated, for example, that family involvement in behaviour change impacts individuals with diabetes at different levels: (1) the patient’s clinical level (improvement of glycosylated haemoglobin and blood pressure);^38^ (2) the psychosocial level (improvement in self-reported depressive symptoms, quality of life and the patient’s perceived supportive resources and physical activity);^39,40,41^ (3) the level of self-management behaviours (improvements in caloric intake, saturated fat, cholesterol and fat consumed);^42^ and (4) the healthcare utilisation level (decrease in emergency room visits and number of hospitalisations).^43^ However, conversely, nonsupportive families are characterised by a lack of respect for the patient’s self-care, attempts to provide unhealthy foods to patients or rigidity in adjusting their routines to the patient’s lifestyle changes; these end up causing low adherence to medication, leading to poor sugar control.^44,45,46^ Health promoters have a key role in determining members of patients’ social networks, including those with positive effects on the adoption of modifiable lifestyle behaviours and those with deterrent roles, in order to prompt positive change by supporting patients to interact differently with each group.

#### Perceived enablers and barriers

Participants in the study revealed enablers at the personal level (being physically fit), in terms of material and human support (availability of equipment, reading materials, guardians and disclosure of the condition to others), and motivation were the main enablers for behaviour change.^37^

Similarly, an early study that assessed perceived enablers and barriers to physical activity among Americans cited social support, positive outcome expectations and programme access as enablers.^47^ Motivation and positive outcome expectations can easily relate to change, as it has been demonstrated that a positive attitude towards a behaviour facilitates behaviour change.

On the other hand, this study observed that addiction, body weakness, lack of money or equipment, loneliness, busyness and being resistant to advice are perceived to play the role of barriers to change. Studies observed health problems, fear of falling and inconvenience, built environment and lack of knowledge about the behavior are also barriers to change.^47^ Other factors include apathy towards diet among male patients; unhealthy diets of friends and family; expected consumption of unhealthy foods in certain situations; the relatively low cost of unhealthy foods; lack of time to plan, shop, prepare and cook healthy foods; lack of facilities to prepare, cook and store healthy foods; widespread presence of unhealthy foods; lack of knowledge and skills to plan, shop, prepare and cook healthy food and lack of motivation to eat healthily.^48^

This implies that planners should be critical of the above-identified enablers and barriers in equipment, social sphere, personal, knowledge and inner components such as motivation to increase the chance of good outcomes in modifiable behaviour change interventions.

### Unexpected results

Our findings showed a clear demarcation between male patients and female patients with regard to the three types of beliefs. The gender narrative, although hidden in the raw data, is quite clear upon analysis.

Regarding the behavioural salient belief type, whereas women used strong words such as ‘back to life’ in describing the advantages of healthy behaviour, men remained relativist in their comments about alcohol and smoking. In terms of normative salient beliefs dichotomy, women focused their attention on their direct social network for approval, while most of the male patients’ focus remained quite poorly defined (except in one male participant who clearly expected approval from his wife, who had been the victim of the effects of his alcohol intake for years). Finally, regarding ‘control salient beliefs’, men relied on external factors (God, known sickness, money, equipment) to launch their behaviour changes, while women relied more on internal factors and those things under their control (having a garden for fruits and vegetables). A previous study demonstrated a link between gender and behaviour change.^49,50^

The implication of dichotomy by gender has to be considered when implementing behaviour change and evaluating its outcomes. This gender consideration in the management seems relevant in Mangochi, which has the largest Muslim population countrywide, and in some rural areas such as Makanjila, Muslims can represent up to 80% of the population. Gender role distribution and social representation are quite strong in Muslim settings.

## Strengths and limitations

The main strength of this study lies in the use of a qualitative approach, which helped to obtain a rich, full and in-depth exploration of the issues surrounding salient beliefs by patients with diabetes and hypertension. However, limitations of the present study include the subjective nature of patients’ responses, the risk of social desirability to please the research team (who are also providers of care) and the fact that the researchers opted to stop at 25 interviews (point of saturation) rather than collect two additional interviews to confirm saturation of data. However, while some consider adding interviews (which will not be considered) a way to confirm data saturation, many authors agree that there is no ‘one-size-fits-all method’ to reach data saturation.^51^ The value of each method used will depend on its operational definition in the study. This lack of supplementary interviews beyond the 25 may have an impact on trustworthiness (transferability). Also, although snowball sampling, used here for selecting some participants, saves time and money as a purposeful sampling technique,^28^ it is appropriate for recruitment in behaviour or lifestyle which deviates from social norms, such as smoking and harmful alcohol use, in a dominant Muslim community such as Mangochi. However, we took care when interpreting the findings, as snowball sampling generates strong fear from participants of disclosing their behaviours to the public or fellow patients, which can affect privacy and confidentiality in their care.^29^

## Recommendations

Further research, informed by the questionnaire from these salient beliefs categories (Appendix 1), should address the following:

- Validation to test their local relevance will be included in research concerning a range of modifiable behaviours in Mangochi, based on the theory of planned behaviour (Appendix 1).
- A quantitative study will determine correlations among relevant variables. In that study, the research seeks to examine whether attitude, subjective norms and perceived behaviour control can predict a patient’s intention to change modifiable risk factors to prevent complications among patients living with diabetes or hypertension in NCD clinics in Mangochi, Malawi.
- The quantitative study will furthermore examine the correlation between these beliefs, categories and patient characteristics, as well as the effectiveness, cost and cost-effectiveness of interventions based on modifiable behaviour change in Mangochi.

For practice:

- The intervention alluded to therein is the implementation of a brief behaviour change intervention informed by the 5 As approach and guided by a motivational interviewing approach, which the research team is planning to test locally in order to estimate its implementation outcomes and later its effectiveness.
- Through such further study and programming, health promotion specialists and counsellors can use the points elucidated in this study to support patients’ behaviour change journeys in Mangochi.

## Conclusion

This is the first formative study conducted in Mangochi, Southern Malawi (even Malawi as a whole), about elicitation of beliefs of laypersons with diabetes and hypertension regarding modifiable health behaviour.

The study suggests that patients believe that changing behaviour leads to healthy life. This can be facilitated by availability of resources and fitness of the body. Generally, the direct social network of patients and caretakers approve of these changes.

Change of modifiable behaviour is possible, even though challenging. For the change to happen, there is a need for patients with diabetes and hypertension who are willing to change modifiable behaviours to screen their social network for support, align the starting behaviour change time with the period when they are physically and mentally fit, devote some financial resources and obtain required equipment to increase the likelihood of success. At the same time, distancing from friends involved directly or indirectly in the unhealthy behaviours, while strengthening relations with their caretakers and relatives also remain key for success.

## Appendix 1

Below is the questionnaire guide used in data collection for the three salient beliefs.

Please take a few minutes to list your thoughts about the following questions. All the questions applied to the following four modifiable risk factors are summarized by the digits 1, 2, 3, and 4, which stand for:

1. eating at least three portions of fruit and vegetables
2. exercising 30 minutes per day, for at least five days per week
3. stopping smoking
4. decreasing harmful alcohol intake

The three groups of questions below are applied mutatis mutandi to the four above modifiable risk behaviors.

When patients with Type 2 diabetes and/or hypertension consult their clinicians [GP, clinical or medical officer],

Behavioral salient beliefs

1. ‘What do you believe would be the advantages of 1, 2, 3, or 4
2. What do you believe would be the disadvantages of 1, 2, 3, or 4
3. What would you like or enjoy about 1, 2, 3, or 4
4. ‘What would you dislike or hate about 1, 2, 3, or 4

Normative beliefs

1. ‘Which individuals or groups of people would approve (i.e. think it was a good idea) of 1, 2, 3, or 4’
2. ‘Which individuals or groups of people would disapprove (i.e. think it was a bad idea) of your 1, 2, 3, or 4

Control beliefs

1. What things (i.e. factors or circumstances) would make you more likely to engage in 1, 2, 3, or 4
2. ‘What things (i.e. factors or circumstances would make you less likely to engage in 1, 2, 3, or 4)
3. Are there any other issues that come to mind when you think about [measuring the blood pressure of a patient with diabetes during a consultation]?

**TABLE 1-A1:** Themes, categories, and their definitions.

Themes	Categories	Definitions or descriptions
Physical and psychological fitness	Normalise the body	Reverted to its initial (anatomic, physiologic, and emotional) state.
	Neutral state of the body	Free of symptoms of diseases, ready to play and work without limitation.
	Free from minor ailments	Decreased likelihood of intermittent minor diseases like flu, headache, general body pain, following behaviour change.
	Well-controlled diabetes or hypertension	Diabetes with no symptoms and normal blood sugar levels, or hypertension with normal blood pressure measurements, less need for medication.
Socially disconnected	Being abandoned	People (fellow smokers and drinkers) distancing from patient, contributing to a feeling of being isolated.
Perceived supportive people	Closed relatives and friends	Receipt of direct assistance in changing behaviour from relatives, friends, partner, or children.
	Fellow patients	Patients waiting at the hall at the clinic routinely providing positive advice in behaviour change.
	Victims of past behaviour	People who have been affected negatively by the behaviour in the past (ex. wife of an aggressive drinker, children of a heavy smoker).
Perceived no supportive people	Fellow drinkers or smokers	People who have shared beers and /or cigarettes with them and who are resisting their change of behaviour.
	Healthy people	People without diabetes/hypertension surrounding a patient.
	Vendors of alcohol or tobacco	Former seller to the patient of alcohol and/or tobacco.
	Unwilling family members	Family members with no or low willingness of assisting with diabetes and/or hypertension.
Perceived enablers	Physical fitness	Having a body without any active symptoms.
	Availability of reading materials	Presence of documents (leaflets, pamphlets, stickers, handouts) on risk behaviour for patients.
	Availability of equipment	Having physical activity equipment, information.
	High motivation	Willingness to change behaviour.
	Having a source of support	Someone who facilitates behaviour change (cook for diets, colleagues to exercise with, etc.).
	Disclosure of the condition	Informing close people (relatives, neighbours, colleagues, etc.) about the diseases and assistance from them in the form of understanding, advice, or supply of equipment.
Perceived barriers	Addiction	Inability to manage to discontinue drinking.
	Lack of money	Those lacking money to cover the basic needs such as foods or family costs (e.g. children’s school fees).
	Refractory to advice	Patient ignores advice related to diabetes and/or hypertension from care providers, fellow patients or influential family members.
	Busy schedule	Working in several businesses or having social and/or civic responsibilities to meet basic needs.
